# Advanced Ceramic Materials with Functional Properties

**DOI:** 10.3390/ma15186439

**Published:** 2022-09-16

**Authors:** Dariusz Bochenek

**Affiliations:** Institute of Materials Engineering, Faculty of Science and Technology, University of Silesia in Katowice, 75 Pułku Piechoty 1a, 41-500 Chorzów, Poland; dariusz.bochenek@us.edu.pl

With the dynamic progress in technology worldwide, the research into new engineering materials applies to a wide range of materials with exciting properties. These materials include improved metal alloys, new types of plastics, ceramics, and composite materials with a wide range of applications, e.g., [1,2]. In terms of modern applications, the most important factors in the field of ceramic materials are high values of dielectric, ferroelectric, piezoelectric, pyroelectric, and magnetic properties obtained in various types of materials, e.g., ferroelectrics, piezoelectrics, pyroelectrics, piezoelastics, multiferroics, ferroelectro-ferromagnetic composites, materials with a perovskite-type structure, doped ceramic materials, lead-free materials, biomaterials, etc. [3,4,5,6]. In recent years, particular emphasis has been placed on experimental and technological research on materials with multiferroic properties for microelectronic and micromechatronic applications [7,8,9]. These studies concern both the design of materials with functional properties obtained in one material [10,11,12] (and joining materials with various properties to form a composite, e.g., ferroelectric with ferrite [13,14,15,16,17,18]) and the design of multi-component materials (e.g., solid solutions) [19,20,21]. Such a connection (with the coupling of the magnetic and electric subsystems) allows for obtaining new material properties, extending the application possibilities of these materials. For example, multiferroic properties can be used in interference sensors sensitive to field changes, during the precise control of electrical and magnetic fields, as well as temperature and pressure, and further in broadband detectors of the far infrared, as tunable multifunction transducers, pyroelectric sensors, oscillators, vibrators, electrostrictive and magnetoelectric transducers, actuators, logic devices (for storing information) and microwave devices [7,8,9,22]. Multiferroic composites with a high magnetoelectric effect and optimal properties are also potential candidates for specific applications in magnetoelectronics or spintronic technology [7,22,23,24]. Appropriate magnetoelectric coupling allows external factors (magnetic field, electric field, stress, or temperature) to control magnetic and electrical properties, which makes it possible to obtain new types of memory in one material [23,24,25,26,27].

Thus, producing various physical properties in one material is a promising way to obtain modern and high-performance engineering materials to obtain their versatile functionality. For example, obtaining a multiferroic ceramic composite, a combination of a material with high dielectric, piezoelectric and ferroelectric properties and a material with high magnetic properties, increases the magnetoelectric effect, which is an important factor in many applications. Various transducers, sensors, and memory elements based on materials with multiferroic properties find newer and more functional applications in microelectronics, cosmology, and high-energy physics [9]. This results in the need for the continuous improvement of this type of material’s production technology to obtain a product with optimal and repeatable physical parameters. Technology improvement is accompanied by the simultaneous search for new kinds of multiferroic materials with exciting properties.

The technology for obtaining ceramic materials at each stage of the technological process is controlled (supported) by a wide range of specialized tests, including thermo-gravimetric (DTA, TG, DTG), X-ray (XRD), and microstructure analysis (SEM, EDS, EPMA, TEM, AFM, STM). In the case of multiferroic ceramic composites and ceramic materials with functional properties, the improvement of physical properties and proper parameters is mainly obtained as a result of optimizing the conditions for their production. These include the conditions of synthesis and sintering, the use of unique sintering methods, the modification of already known technological methods, the appropriate selection and optimization of technological methods for a specifically designed multiferroic composite composition, as well as the appropriate modification of the base composition. All of the above activities in ceramic technology processes are used to enhance the properties and broaden the application of ceramic materials, e.g., [7,8,9,28]. Specialist tests of the final properties of ceramic materials and their reliability and stability (over time and under changing conditions of use) are used to determine new application possibilities for materials with functional properties. These include, for example, testing of the ferroelectric, dielectric, piezoelectric, pyroelectric, piezoelastic, magnetic, and magnetoelectric properties, Raman spectroscopy, impedance tests, electric conductivity, etc. [29,30,31].

The experimental and research issues relating to modern materials engineering in materials with functional properties presented above constitute the thematic scope of this Special Issue, entitled “Advanced Ceramic Materials with Functional Properties”. The main objective of this Special Issue is to publish outstanding papers presenting advanced research in the field of ceramic materials and ceramic composites with functional properties and broad applications.
